# 5′ DREDGE: Direct Repeat-Enabled Downregulation of Gene Expression via the 5′ UTR of Target Genes

**DOI:** 10.3390/cells14120866

**Published:** 2025-06-08

**Authors:** Sagar J. Parikh, Heather M. Terron, Luke A. Burgard, Dylan D. Butler, Frank M. LaFerla, Shelley Lane, Malcolm A. Leissring

**Affiliations:** 1 Institute for Memory Impairments and Neurological Disorders, University of California, Irvine, CA 92697, USA; 2 Department of Neurobiology and Behavior, University of California, Irvine, CA 92697, USA

**Keywords:** CRISPR, direct repeat, DREDGE, endoribonuclease, gene regulation

## Abstract

Despite the availability of numerous methods for controlling gene expression, there remains a strong need for technologies that maximize two key properties: selectivity and reversibility. To this end, we developed a novel approach that exploits the highly sequence-specific nature of CRISPR-associated endoribonucleases (Cas RNases), which recognize and cleave short RNA sequences known as direct repeats (DRs). In this approach, referred to as DREDGE (direct repeat-enabled downregulation of gene expression), selective control of gene expression is enabled by introducing one or more DRs into the untranslated regions (UTRs) of target mRNAs, which can then be cleaved upon expression of the cognate Cas RNase. We previously demonstrated that the expression of target genes with DRs in their 3′ UTRs are efficiently controlled by the DNase-dead version of Cas12a (dCas12a) with a high degree of selectivity and complete reversibility. Here, we assess the feasibility of using DREDGE to regulate the expression of genes with DRs inserted in their 5′ UTRs. Among the five different Cas RNases tested, Csy4 was found to be the most efficient in this context, yielding robust downregulation with rapid onset in doxycycline-regulatable systems targeting either a stably expressed fluorescent protein or an endogenous gene, both in a fully reversible manner. Unexpectedly, dCas12a was also found to be modestly effective despite binding essentially irreversibly to the cut mRNA on its 5′ end and thereby boosting mRNA levels. Our results expand the utility of DREDGE as an attractive method for regulating gene expression in a targeted, highly selective, and fully reversible manner.

## 1. Introduction

Direct repeats (DRs) are short sequences of RNA that are recognized and cleaved by CRISPR-associated endoribonucleases (Cas RNases) in a highly sequence-specific manner [1,2,3]. The high degree of specificity of Cas RNases for their cognate DRs suggests that this interaction could be exploited to selectively target and cleave specific mRNAs incorporating one or more DRs. Confirming this, we recently demonstrated that Cas RNases can efficiently downregulate target genes with DRs incorporated into their 3′ untranslated regions (UTRs) [4]. This approach, referred to as 3′ DREDGE (direct repeat-enabled downregulation of gene expression), depends on the fact that removal of the poly(A) tail from mRNAs triggers rapid degradation via deadenylation-dependent mRNA decay [4,5]. We found that 3′ DREDGE could be implemented by multiple Cas RNases, the most efficient being the DNase-dead version of Cas12a (dCas12a) [4,6]. 3′ DREDGE proved to be highly effective, resulting in >90% downregulation of the target genes in a manner that was rapid, highly selective, and completely reversible [4].

**Figure 1:**
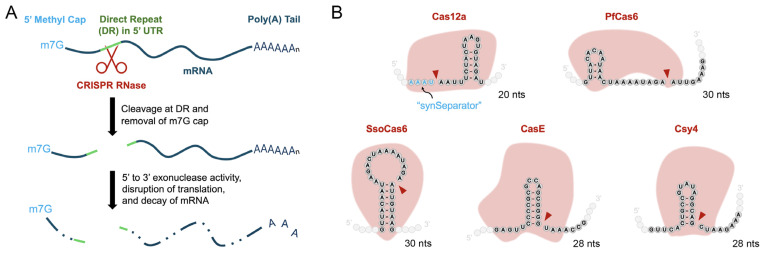
Cas RNases screened for suitability for 5′ DREDGE. (**A**) Cartoon illustrating the basic principles of 5′ DREDGE. DRs (green) placed into the 5′ UTR of an mRNA are cleaved by Cas RNases (red), resulting in the removal of the 7-methylguanosine (m7G) 5′ cap, which in turn impairs translation by multiple mechanisms and also triggers mRNA degradation via 5′-3′ exonuclease activity (see text). (**B**) DRs for the five Cas RNases tested in this study. Cleavage sites are indicated with red arrows and the sizes in nucleotides (nts) are shown. Note the “synSeparator” (AAAU) upstream of the 5′ end of the Cas12a DR.

In the present study, we evaluated the effectiveness of DREDGE in controlling the expression of genes with DRs incorporated into their 5′ UTRs. In this variation, dubbed 5′ DREDGE, cleavage of the DRs by the Cas RNase results in separation of the bulk of the mRNA from the 5′ portion containing the 7-methylguanosine 5′ cap (see Figure 1A) [7]. Removal of the 5′ cap, in turn, disrupts translation and triggers mRNA decay via any of several mechanisms. Translation is impaired both because the 5′ cap normally facilitates export of mRNAs from the nucleus and also because it is critical for interactions with translation initiation factors [8,9]. Uncapped mRNA also decays rapidly through 5′-3′ exonucleolytic degradation, which is normally blocked by the 5′ cap [10,11].

Here, we compared the efficacy of 5′ DREDGE implemented with five different Cas RNases and their cognate DRs and identified Csy4 (also known as Cas6f) as the most effective in a transient transfection paradigm [12]. dCas12a also performed relatively well in this paradigm so it was evaluated in parallel. In stable cell lines expressing destabilized green fluorescent protein (GFPd2) with cognate DRs in their 5′ UTRs, efficient downregulation was achieved by Csy4, while more modest reductions were obtained using dCas12a. dCas12a expression was found to dramatically *increase* GFPd2 mRNA due to its essentially irreversible binding to the downstream 5′ end of the cleaved mRNA, thus explaining its poor performance. Csy4 was subsequently shown to efficiently downregulate an endogenous gene after introduction of a DR into its 5′ UTR via CRISPR-Cas. 5′ DREDGE achieved >90% downregulation of the endogenous gene and—crucially—in a manner that was fully reversible even after several months of continuous downregulation. Our results establish 5′ DREDGE as an effective means to tightly control gene expression, with multiple advantages over current technologies. 

## 2. Materials and Methods

### 2.1. Cloning of Expression Constructs

#### 2.1.1. Vectors for Transient Transfection Experiments

The vector expressing destabilized GFP (GFPd2) was generated by modifying Addgene plasmid #14760 [13] to include a puromycin resistance cassette, as described [4]. After linearization by digestion at an SmaI site within the 5′ UTR of the GFPd2 ORF, de novo synthesized DNA fragments (Integrated DNA Technologies (IDT), San Diego, CA, USA) encoding the cognate DRs for 5 different Cas RNases were inserted using NEBuilder technology (New England Biolabs (NEB), Beverly, MA, USA). ORFs encoding dCas12a, with or without 2 nuclear localization signals, were PCR-amplified from pSLQ10844 (Addgene Plasmid #183956 [6]). De novo DNA synthesis (IDT, San Diego, CA, USA) was used to generate mammalian codon-optimized gBlock DNA sequences identical to those in Campa et al. [14], which encoded ORFs for CasE, Csy4, SSoCas6, and PfCas6 and featured a FLAG tag either alone or together with an NLS at the N-terminus. A vector co-expressing mCherry via an internal ribosomal entry site, pICherryNeo (Addgene Plasmid #52119; gift of Dr. Vignali), was linearized with XbaI and the gBlocks were inserted using NEBuilder (NEB, Beverly, MA, USA). Next-generation DNA sequencing was performed on all constructs to verify their integrity (Azenta Life Sciences, Burlington, MA, USA).

#### 2.1.2. Vectors for Dox-Regulatable Co-Expression of Cas RNases, mCherry, and Neo^r^

Vectors for inducible expression of Cas RNases (or no RNase) along with mCherry (mCh) with three C-terminal NLSs and neomycin/G418 resistance (Neo^r^) were generated by first creating the “No-RNase” parental vector, pMT_mCh_NeoR_pT1, which contained the pTet-One [15] system, as described in [4] (Takara Bio USA, Inc., San Jose, CA, USA). PCR was used to amplify the ORF for Csy4 containing a FLAG tag and an NLS from the version cloned into pICherryNeo; for dCas12a, we amplified a codon-optimized ORF (with two C-terminal NLSs) from pSLQ10875 (Addgene Plasmid #183962 [6]). Both ORFs were subsequently cloned into the pMT_mCh_NeoR_pT1 vector, linearized with NcoI and SacI, using NEBuilder (NEB, Beverly, MA, USA).

### 2.2. Cell Culture

Mouse embryonic fibroblasts (MEFs; wild-type, SV40-immortalized; ATCC Cat #CRL-2907, Manassas, VA, USA) were cultured under standard conditions in DMEM supplemented with 10% Tet System Approved Fetal Bovine Serum (Takara Bio USA, San Jose, CA, USA), GlutaMAX^®^, streptomycin (100 µg/mL) and penicillin (100 U/mL).

### 2.3. FACS and Flow Cytometry

To compare the suitability of the different Cas RNases for 5′ DREDGE, MEFs were cotransfected with GFPd2- and Cas RNase-expressing vectors using the Amaxa Nucleofector II electroporation system (Lonza Bioscience, Bend, OR, USA) and cultured overnight. Immediately prior to collection, a Zeiss Axiovert 200 m fluorescent microscope was used to collect representative fluorescent images (Zeiss, Dublin, CA, USA). For flow cytometry, a BD LSRFortessa™ X-20 Cell Analyzer was used to quantify the GFP and mCherry fluorescence (BD BioSciences, Franklin Lakes, NJ, USA). For the generation of clonal cell lines, a BD FACSAria™ II Cell Sorter was used to sort individual cells into multiwell plates (BD BioSciences, Franklin Lakes, NJ, USA).

### 2.4. Real-Time PCR

After growth for three days with or without Dox (1 µg/mL), double-stable cell lines expressing either dCas12a or Csy4 together with GFPd2 containing the cognate DRs were homogenized with QIAshredder™ and total mRNA was subsequently purified using the RNeasy Plus Mini Kit (QIAGEN, LLC, Germantown, MD, USA). Reagents from the SingleShot™ SYBR^®^ Green One-Step Kit were used to perform SYBR green-based real-time PCR (RT-PCR) on a CFX96 Touch Real-Time PCR Detection System (Bio-Rad, Hercules, CA, USA). The primer pairs (Fwd, Rev) used were GFP-A (ATGTCTTGTGCCCAGGAGAG, GTGGTATTTGTGAGCCAGGG) and GAPDH (CCCACTCTTCCACCTTCGAT, GAGTTGGGATAGGGCCTCTC).

### 2.5. Insertion of a Csy4 DR into the 5′ UTR of the CTSD Gene

The Csy4 DR was introduced into the 5′ UTR of the cathepsin D (CatD) gene (*CTSD*) in MEFs using a de novo synthesized targeting construct containing: (1) the Csy4 DR within exon 1 of *CTSD*; (2) a cassette expressing puromycin resistance (Puro^r^) under the control of the PGK promoter within the first intron, flanked by FRT sites to permit its removal with Flp-recombinase; and (3) ~600 bp 5′ and 3′ homology arms (see Supplementary Figure S1B; GenScript USA, Piscataway, NJ, USA). For CRISPR-Cas, cells were transfected as above with a mixture comprising (1) recombinant Alt-R A.s. Cas12a (Cpf1) *Ultra* Nuclease; (2) two synthetic gRNAs (GGGAGUCUUCAUGGUCGCGG and ACCUGGAGUGCCGCGUGCUC); and (3) 0.5 µg of a PCR product amplified from the targeting construct generated using two oligonucleotides (Fwd, C*A*TTGCTACATTATGGAATG-TGCATG; Rev, C*A*CCAAGACCTCATCTAGCAC; asterisks indicate the placement of phosphorothioate bonds that were incorporated to inhibit degradation) (IDT, San Diego, CA, USA). After selection of Puro-resistant cells and the identification of positive clones, the Puro^r^ cassette was removed by transfecting the cells with pCAG-Flpe:GFP (Addgene Plasmid #13788) [13]. Two days after transfection, individual GFP-positive were isolated by cell sorting, and then screened by PCR to confirm removal of the Puro^r^ cassette.

### 2.6. CatD Activity Assays

CatD activity assays were conducted on cell lysates by monitoring time-dependent increases in fluorescence (328 nm excitation, 393 nm emission) produced by proteolytic digestion of a fluorogenic peptide, Mca-GKPILFFRLK(Dnp)-R-NH_2_ (InnoPep, Inc., San Diego, CA, USA) [4,15,16]. The protein concentrations in the lysates, quantified by relative absorbance at 280 nm with a NanoDrop^®^ ND-1000 UV-Vis Spectrophotometer, were used to normalize activity data (Thermo Fisher Scientific, Waltham, MA, USA).

## 3. Results

### 3.1. Selection and Screening of Candidate Cas RNases for 5′ DREDGE

To screen for Cas RNases suitable for the 5′ DREDGE approach (Figure 1A), we selected five for investigation (Figure 1B). Cas12a from *Lachnospiraceae bacterium* (also known as Cpf1; Figure 1B) was chosen because it proved to be the top-performing Cas RNase in 3′ DREDGE [4]. Cas12a is atypical because it also possesses DNase activity; hence, we utilized a DNase-dead version, specifically the “hyperdCas12a” variant [6]. On the 5′ end of the Cas12a DR, we also included a “synthetic separator” (synSeparator)—AAAU—that promotes cleavage by dCas12a [17] (Figure 1B). PfCas6 from *Pyrococcus furiosus* and SsoCas6 from *Sulfolobus solfataricus* (Figure 1B) were selected because they are “multiple-turnover enzymes” in contradistinction to Cas RNases that are unable to process additional RNA molecules because they remain tightly bound to the DR after cleavage, which are referred to as “single-turnover” enzymes [2]. Finally, we chose Csy4 (also known as Cas6f) from *Pseudomonas aeruginosa* and CasE (also named EcoCas6e) from *Escherichia coli* because these were the best-performing Cas RNases from among nine tested in a recent study that introduced their cognate DRs into mRNAs [14].

The cognate DRs of the five Cas RNases (Figure 1B) are all quite short, comprising ≤30 nucleotides, but highly varied in terms of primary nucleotide sequence (Figure 1B). Of note, only Cas12a cleaves outside the DR, at the 5′ end, unlike the other Cas RNases that all cleave within the DRs at positions near their 3′ ends.

**Figure 2:**
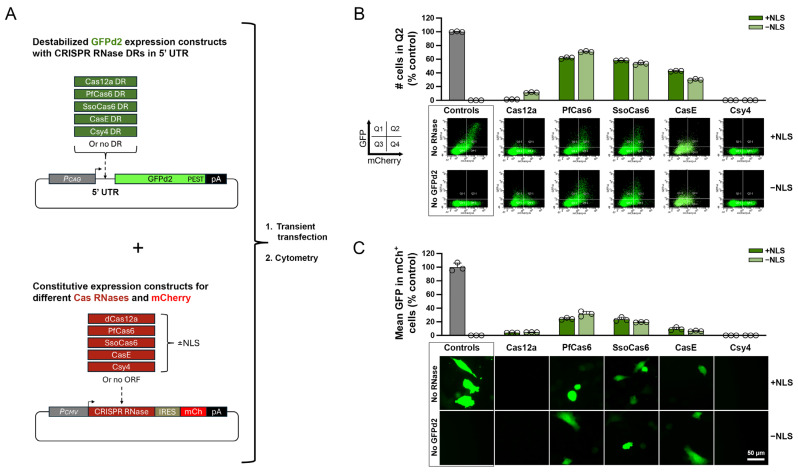
Comparative efficacy of 5′ DREDGE implemented with five different Cas RNases and their cognate DRs. (**A**) Overview of the construction of the vectors encoding GFPd2 with individual cognate DRs (green) in the 5′ UTR (top) and vectors co-expressing different Cas RNases (red) together with mCherry (bottom). Constructs lacking a DR or RNase served as controls. (**B**) Cell cytometry results from MEFs transiently transfected with the constructs in (**A**). Depicted are the percentages of cells in Q2 relative to controls (top), which were quantified from log-log plots of GFP vs. mCherry RFU values (*n* = 3 replicates), with representative plots for each condition provided (bottom). (**C**) GFP intensity in mCherry-positive cells, normalized to controls, derived from the RFU plots shown in (**B**), together with representative images of GFP fluorescence in cells from the various conditions (bottom), which were acquired immediately prior to cytometry.

To assess the suitability of the five selected Cas RNases for 5′ DREDGE, we developed a two-part model system for co-expression of (1) a target gene with an appropriate DR in its 5′ UTR and (2) the corresponding Cas RNase, which utilizes GFP and mCherry fluorescence, respectively, as convenient fluorescent markers for the expression of the two components (Figure 2A). The first part consisted of vectors encoding a destabilized form of GFP (GFPd2) with a very short (~2-h) half-life (t_1/2_) [13]. For each of the five Cas RNases we screened, one copy of the cognate DR was introduced into the 5′ UTR of this construct. The unmodified vector served as a “No-DR” control (Figure 2A, top). The second part of this system relied on a vector expressing mCherry via an internal ribosomal entry site, into which was cloned ORFs for each of the five Cas RNases, including versions without or with nuclear localization signals (NLSs), thus generating 10 different Cas RNase expression constructs plus the parent vector, which served as a “No-RNase” control (Figure 2A, bottom).

To assess the relative efficacy of the different RNases, MEFs were cotransfected with each Cas RNase/mCherry construct along with the GFPd2 construct harboring the cognate DR in its 5′ UTR. One day later, the transfected cells were analyzed by cell cytometry. Log-log plots of mCherry and GFP fluorescence on the X- and Y-axes, respectively, were generated and subdivided into four quadrants by using untransfected cells to establish boundaries for GFP and mCherry fluorescence (Figure 2B). Control cells cotransfected with No-DR GFPd2 and No-RNase vectors exhibited high levels of both GFP and mCherry fluorescence, resulting in large numbers of cells appearing in the upper right quadrant (Q2) of the RFU plot (see Figure 2B). Relative to these controls, the expression of each of the five Cas RNases together with the GFPd2 variants containing the respective 5′ UTR DRs triggered substantial decreases in the relative proportion of cells appearing in Q2 (Figure 2B). The efficacy of the Cas RNases was even more substantial when assessed in terms of the mean GFP fluorescence levels in mCherry-positive cells (e.g., cells in Q2 and Q4; Figure 2C). Csy4 outperformed all the other Cas RNases as assessed by both of these metrics, resulting in essentially no cells present in Q2 (Figure 2B) and mean GFP RFU levels comparable to control cells expressing no GFPd2 (Figure 2C). dCas12a also performed well, albeit with some notable differences. In particular, while essentially no cells were present in Q2 for cells expressing dCas12 with two NLSs, the version lacking an NLS did not achieve complete downregulation (Figure 2B). Also, both versions of dCas12a failed to achieve complete downregulation, as assessed by the mean GFP fluorescence (Figure 2C). The remaining Cas RNases performed comparatively poorly (Figure 2B,C), so we elected to conduct further analyses exclusively with dCas12a and Csy4 with NLS sequences.

### 3.2. Dox-Regulatable Gene Expression by 5′ DREDGE Using dCas12a and Csy4

**Figure 3:**
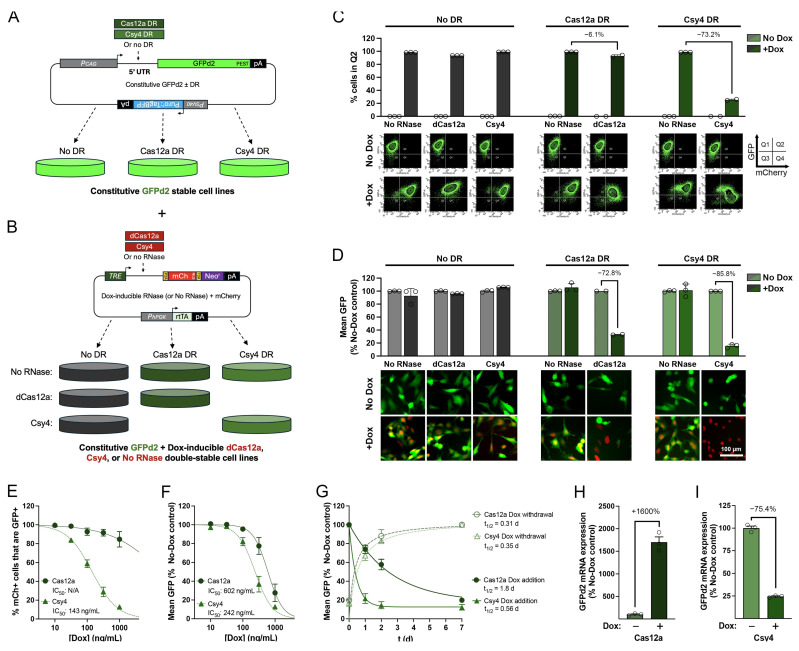
Characterization of 5′ DREDGE using an inducible system. (**A**) Cartoon depicting the generation of clonal cell lines constitutively expressing GFPd2 with a single Cas12a or Csy4 DR in the 5′ UTR (or no DR as a control). (**B**) Generation of double-stable lines for inducible Cas RNase expression. The lines in (**A**) were used to generate lines that also stably express either dCas12a or Csy4 (or no RNase as a control) in a Dox-regulatable manner. (**C**,**D**) Performance of the double-stable cell lines from (**B**), assessed using the percentage of cells in Q2 (**C**) and mean GFP RFU (**D**) in the absence or presence of Dox (1 µg/mL). Data are shown as the mean ± SEM normalized to Dox-treated No-RNase controls; *n* = 2–3 per condition. (**E**,**F**) Dox dose–response experiments with double-stable cell lines inducibly expressing dCas12a or Csy4. Graphs depict responsiveness to a range of concentrations of Dox, quantified in terms of the percentage mCherry-positive cells that were also GFP-positive (**E**) or the mean GFP RFU in all cells (**F**). Mean IC_50_ values are shown. Data are shown as the mean ± SEM for 2–3 replicates per condition, normalized to values in the absence of Dox for each line. (**G**) Temporal dynamics of GFPd2 expression in cell lines inducibly expressing either dCas12a or Csy4 following addition (solid lines) or withdrawal (dashed lines) of Dox (1 µg/mL). Data are shown as the mean ± SEM for 2–3 independent experiments, normalized to No-Dox controls. Mean t_1/2_ values are indicated. (**H**,**I**) Relative expression of GFPd2 mRNA in the absence versus the presence of Dox (1 µg/mL) in double-stable cell lines instantiating 5′ DREDGE with Cas12a (**H**) and Csy4 (**I**). Note the dramatic increase in GFPd2 mRNA levels triggered by dCas12a expression (**H**). Data are mean ± SEM for 3 independent experiments, expressed as a percentage of No-Dox controls.

To assess the ability of 5′ DREDGE to regulate gene expression in an inducible manner, we generated double-stable cell lines as follows. First, single clonal cell lines stably expressing GFPd2 were generated, including variants lacking any DR, as a control, or containing a single Cas12a or Csy4 DR in the 5′ UTR (Figure 3A). Second, these stable GFPd2-expressing lines were used to generate double-stable lines also expressing dCas12a or Csy4 (or no RNase) in a doxycycline (Dox)-regulatable manner, which was implemented using the Tet-One™ system (Takara Bio USA, Inc., San Jose, CA, USA; Figure 3B) [18]. The Cas RNase-expressing constructs were designed to co-express both mCherry, as a facile marker for expression, and neomycin resistance, as a means to select for the dual-stable lines (Figure 3B). As expected, the No-DR GFPd2 controls exhibited high levels of GFP fluorescence independent of Cas RNase expression (Figure 3C,D). The No-RNase control cells also behaved as predicted, with no reduction in GFPd2 expression triggered by Dox treatment, regardless of the presence or absence of DRs in the 5′ UTR (Figure 3C,D, middle). Unlike the substantial downregulation achieved with dCas12a in the transient transfection paradigm (Figure 2B,C), Dox-induced expression of dCas12a in the GFPd2 line with its cognate DR elicited only a very modest (~6%) reduction in the number of cells in Q2. In marked contrast, relative to the same parental lines expressing No-RNase mCherry controls, expression of Csy4 in the GFPd2 line with the Csy4 DR triggered substantial reductions in the number of GFP-positive cells in Q2 (73.2%) (Figure 3C, right). In terms of mean GFP fluorescence (Figure 3D), the cells with Dox-induced dCas12a or Csy4 expression exhibited 72.8% and 85.5% reductions, respectively (Figure 3D, middle and right). Thus, whereas Csy4 performed well for 5′ DREDGE as assessed by both metrics, dCas12a appeared to achieve a uniform but relatively modest decrease in GFP fluorescence in the majority of GFP-expressing cells, with very few cells exhibiting complete downregulation.

To more quantitatively assess the relative efficacy of these two Cas RNases in 5′ DREDGE, we conducted dose–response experiments with Dox (Figure 3E,F). Using the percent of mCherry-positive cells (Q2 + Q4) present in Q2 as a metric, Csy4 yielded an IC_50_ value of 143 ng/mL Dox (Figure 3E); similar results were obtained using the mean GFP fluorescence in all cells, yielding an IC_50_ of 242 ng/mL Dox (Figure 3F). dCas12a performed less well, yielding an IC_50_ of 602 ng/mL Dox using the latter metric (Figure 3F); using the former metric, however, its performance was so poor as to preclude calculation of an IC_50_ value (Figure 3E).

Next, we investigated the kinetic performance of 5′ DREDGE implemented with the two Cas RNases by carrying out time courses of responsiveness after the addition or removal of Dox (Figure 3G). Consistent with the results obtained for 3′ DREDGE [4], the 5′ DREDGE approach exhibited notably fast kinetics, particularly for Csy4. The t_1/2_s for induction of downregulation by addition of Dox were 0.56 d for Csy4 and 1.8 d for dCas12a; in contrast, the t_1/2_s for restoration of activity after withdrawal of Dox were both remarkably fast: 0.31 and 0.35 d, respectively (Figure 3G).

Finally, we quantified GFPd2 mRNA levels in these double-stable lines in the absence vs. the presence of Dox using real-time PCR (RT-PCR). Addition of Dox to the Csy4 line resulted in a 75% reduction in GFPd2 mRNA levels (Figure 3I), a figure in excellent agreement with the magnitude of the reduction in GFPd2 expression quantified by cell cytometry (Figure 3C,D). In marked contrast, induction of dCas12a expression by Dox resulted in a remarkable 16-fold *increase* in GFPd2 mRNA levels (Figure 3H). This intriguing finding helps to explain the comparatively poor performance of this Cas RNase in 5′ DREDGE, as will be discussed in greater depth in Section 4.

### 3.3. Downregulation of an Endogenous Gene by 5′ DREDGE

**Figure 4:**
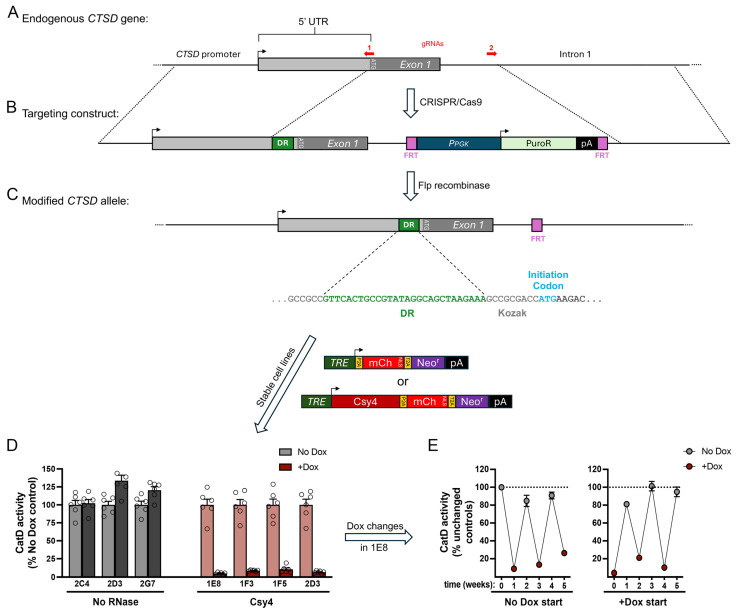
Control of an endogenous gene with 5′ DREDGE. (**A**) Genomic structure of the 5′ end of murine *CTSD*. (**B**) Design of the targeting construct used to introduce a single Csy4 DR into the 5′ UTR of *CTSD* together with a Puro^r^ resistance cassette within the first intron. Note the presence of two FRT sites flanking the Puro^r^ resistance cassette (purple). (**C**) Structure of the modified *CTSD* allele after removal of Puro^r^ resistance cassette with Flp-recombinase. The complete sequence of the Csy4 DR is shown (green), highlighting its location relative to the initiation codon (blue). One cell line with sequence-verified insertion of the Csy4 DR was transfected with the indicated constructs to generate individual stable lines expressing Csy4 or no RNase in a Dox-regulatable manner. (**D**) CatD activity in the latter stable lines in the presence of Dox (1 µg/mL) relative to no Dox. Data are shown as the mean ± SEM; *n* = 5–6. (**E**) CatD activity in stable line 1E8 subjected to repeated cycles of Dox addition and withdrawal on a weekly basis. Graphs depict CatD activity in cells initially grown in the absence (left) or presence (right) of Dox (1 µg/mL), normalized to unchanged controls. Data are shown as the mean ± SEM; *n* = 8 replicates per condition.

The utility of 5′ DREDGE would be greatly enhanced if it could be used to regulate the expression of an endogenous gene. To test this, we elected to target the murine cathepsin D (CatD) gene (*CTSD*), which we regulated successfully via two other technologies, including 3′ DREDGE [4,16]. We used a multi-step approach to add a Csy4 DR into the 5′ UTR of *CTSD* and then created a stable line expressing Csy4 in a Dox-inducible manner (Figure 4; Supplementary Figure S1). First, we used CRISPR-Cas9 to insert a targeting construct containing the Csy4 DR (positioned immediately upstream of the Kozak consensus sequence) together with a Puro^r^ cassette flanked by FRT sites (positioned within the first intron) (Figure 4B; Supplementary Figure S1B). Second, after identification of a positive clone homozygous for this knockin construct (Supplementary Figure S1), we removed the Puro^r^ cassette in this line using Flp-recombinase (Figure 4C). Finally, after identification of a cell line with successful removal of the Puro^r^ cassette, we used the Dox-regulatable constructs expressing either Csy4 or no RNase to generate stable cell lines (Figure 4D). Multiple individual clones for each line were generated, which behaved as expected: as assessed by CatD activity assays, addition of Dox produced substantial (>90%) decreases in CatD levels in cells expressing Csy4 in four different cell lines, whereas no decreases were triggered by Dox in the No-RNase controls (Figure 4D). Notably, even after continuous treatment with Dox for as long as 2 months during the selection of these lines, the CatD activity levels in the inducible Csy4 lines were indistinguishable from the No-RNase controls after removal of Dox. Finally, to more thoroughly demonstrate the reversibility of 5′ DREDGE, we subjected one of the Csy4-expressing clones to multiple successive alternating treatments without or with Dox, spaced approximately one week apart; as shown in Figure 4E, this line exhibited complete reversibility even after five rounds of Dox changes.

## 4. Discussion

Our group conceived of the general approach of DREDGE as a novel means to achieve targeted downregulation of genes in a fully reversible manner, with the explicit goal of evaluating its suitability for in vivo applications [15,19,20]. As our recent analysis demonstrated [4], 3′ DREDGE implemented with dCas12a yields robust downregulation of target genes with rapid kinetics and, crucially, in a manner that is both highly selective and completely reversible. The present study extends this work, establishing that DREDGE can also be implemented by incorporating a Cas RNase DR within the 5′ UTR of target genes. In this case, Csy4 was found to be the most effective for 5′ DREDGE among the five Cas RNases tested, performing comparably to 3′ DREDGE implemented with dCas12a when used to target both a stably expressed protein and an endogenous gene [4].

Unexpectedly, our initial screening of candidate Cas RNases using a transient transfection paradigm suggested that dCas12a might also be effective for 5′ DREDGE. This was surprising given that dCas12a is known to remain tightly bound to RNA after cleavage on the 5′ end of the DR (Figure 1B) [21,22,23]. Further assessment, however, revealed that dCas12a performed poorly in stable cell lines compared both to Csy4 and to transient transfection with dCas12a. Moreover, consistent with the essentially irreversible interaction between dCas12a and its cognate DR, the mRNA levels of the target gene in stable cells were markedly increased when dCas12a expression was induced for several days. These findings suggest, on the one hand, that binding of dCas12a to the cognate mRNA disrupts translation of the bound mRNA, which might occur due to any of several mechanisms. For instance, because the 5′ cap is critical for export of mRNA to the cytosol [9], it seems reasonable that removal of the cap by dCas12a impairs this process. This explanation is supported by the observation that dCas12a without an NLS performed less well in the transient transfection paradigm compared to the version with two NLSs (c.f., Figure 2B). Another plausible mechanism may involve reduced translation of the dCas12a-bound mRNA due to impaired interaction with translation initiation factors that depend upon the presence of the 5′ cap [8]. Finally, we speculate that the dCas12a protein itself might also inhibit translation, possibly through steric blockade of the mRNA from productive engagement with the ribosome. On the other hand, binding of the mRNA also prevents decay of the mRNA, increasing its levels over time and thus acting as a countervailing influence on the degree of translation of the target gene. In the transient transfection paradigm, assessed just 24 h later, mRNA accumulation likely did not occur to the same extent, thus explaining the enhanced downregulation of the target gene by dCas12a in this context. Most or all of these mechanisms are likely to be operating simultaneously and to varying degrees. While they help to account for the unexpected behavior of dCas12a, given the poor performance of this Cas RNase in 5′ DREDGE, there seems to be scant rationale for further elucidation of the underlying mechanisms.

It is instructive to compare the relative merits of 5′ DREDGE implemented with Csy4 to 3′ DREDGE implemented with dCas12a, specifically the version featuring a single Cas12a DR [4]. The two approaches were comparable in terms of the degree of downregulation achieved in the presence of Dox (typically >85%). Sensitivity to Dox was also similar, with IC_50_s of 242 vs. 187 ng/mL obtained for 5′ DREDGE and 3′ DREDGE, respectively (comparable to the EC_50_ for RNase expression itself [4]). In terms of kinetics, both approaches yielded similar t_1/2_s upon Dox addition (0.61 vs. 0.56 d), with Csy4 showing a somewhat faster recovery of gene expression upon Dox withdrawal (0.31 vs. 0.52 d, respectively) [4]. Perhaps the most significant distinction between the two approaches relates to their respective selectivity for the gene of interest. Zhang and colleagues recently performed RNA-seq to assess off-target effects of Csy4 expression in HEK cells [24]. Csy4 expression yielded a relatively high percentage (~5.4%) of differentially expressed genes (DEGs), which compares unfavorably to the 1.9% DEGs (between two separate stable cell lines) obtained in our own RNA-seq analysis of dCas12a expression in MEFs [4]. Crucially, however, Zhang and colleagues developed a drug-activatable “split” version of Csy4 that resulted in a far lower percentage (1.5%) of DEGs [24]. Implementing 5′ DREDGE with this drug-activatable version of Csy4, therefore, may reduce the risk of off-target effects while providing a novel method for drug-inducible downregulation.

## 5. Conclusions

Our results establish 5′ DREDGE as an effective method for achieving reversible downregulation of target genes, with several unique advantages. Only minimal modification to the 5′ UTR of the target gene is necessary—the addition of just 28 nucleotides in the case of the Csy4 DR—thus minimizing the potential for interference with transcription or translation or interactions with *trans*-acting elements such as microRNAs [25]. More-over, in contrast to RNAi and CRISPRi, which rely on RNA:RNA and RNA:DNA complementarity that can tolerate mismatches to varying degrees, 5′ DREDGE relies instead on a unique protein–RNA interaction between non-mammalian *cis*- and *trans*-elements. In summary, our findings demonstrate that 5′ DREDGE constitutes a viable alternative for achieving the controlled regulation of target genes, one that is particularly well suited to applications requiring efficient downregulation and complete reversibility.

## Data Availability

Data are contained within the article and Supplementary Material. Original, raw fluorescence microscopy image files were deposited at the Open Science Framework repository, available at: www.doi.org/10.17605/OSF.IO/EUV7H (accessed on 24 April 2025).

## Supplementary Materials

The following supporting information can be downloaded at: https://www.mdpi.com/article/10.3390/cells14120866/s1, Figure S1: Genotyping strategy and results confirming the successful introduction of one Csy4 DR into the 5′ UTR of murine CTSD via CRISPR-Cas9.
