# Supplementary material for: 5′ DREDGE: Direct Repeat-Enabled Downregulation of Gene Expression via the 5′ UTR of Target Genes
Source: Cells. 2025 Jun 8;14(12):866. doi: 10.3390/cells14120866 (PMC12191184; doi:10.3390/cells14120866)
Supplement: Supplementary file 1 [file cells-14-00866-s001.zip › cells-3635897-supplementary.pdf]

Supplemental Materials
for

# 5' DREDGE: Direct Repeat-Enabled Downregulation of Gene Expression via the 5' UTR of Target Genes

**Sagar J. Parikh[1], Heather M. Terron[1], Luke A. Burgard[1,2], Dylan D. Butler[1,2], Frank M. LaFerla[1,2], Shelley Lane[1] and Malcolm A. Leissring[1,*]**

[1] Institute for Memory Impairments and Neurological Disorders, University of California, Irvine, Irvine, CA 92697, USA
[2] Department of Neurobiology and Behavior, University of California, Irvine, Irvine, CA 92697, USA

[*] Correspondence: m.leissring@uci.edu

## Contents


| pp. | Fig./Table | Title |
|---|---|---|
| 2 | Fig. S1 | Genotyping strategy and results confirming the successful introduction of one Csy4 DR into the 5' UTR of murine CTSD via CRISPR-Cas9. |

# Supplementary Figure S1.

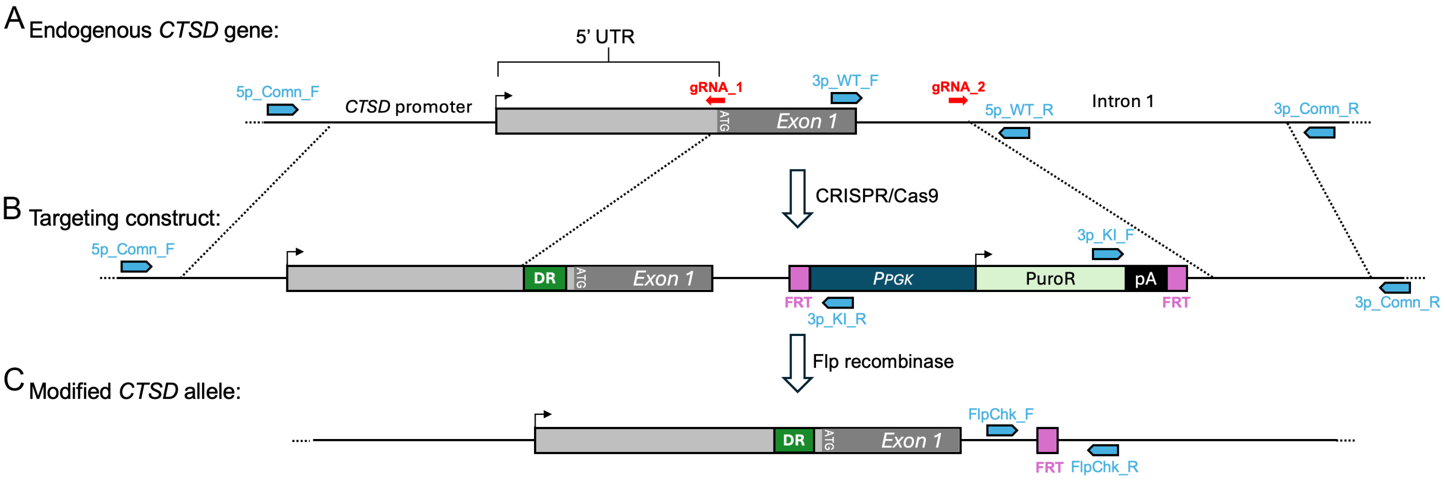


D gRNA design and alterations to knock-in insert:

| | gRNA_1 | gRNA_2 |
|---|---|---|
| **gRNA sequence** | **GGGAGTCTTCATGGTCGCGG** | **ACCTGGAGTGCCGCGTGCTC** |
| **Endog. Sequence (top strand)** | ...CCGCCGCGACCATGAAGACTCCC... | ...ACCTGGAGTGCCGCGTGCTCCGG... |
| **A.A. sequence** | MetLysThrPro… | N/A |
| **KI insert sequence** | …**GAAA**GCCGCGACCATGAAGACTCCC... | deleted and replaced w/ FRT site |

E Genotyping primers:

| Primer Name | Primer Sequence |
|---|---|
| 5p_Comn_F | CCTTGTATCAATAGATGCATAGTC |
| 5p_WT_R | CCTACTGTACCCTCCTGTTC |
| 5p_KI_R | GAATAGGAACTTCGAACGCG |
| 3p_Comn_R | CTCTACTCCCGCAGCCTACG |
| 3p_WT_F | GTCCTCCTTCGCGATTATCAGGTG |
| 3p_KI_F | GCTCGACTAGAGCTTGCGGAAC |
| FlpChk_F | GTCTTGCTGCTCATTCTCG |
| FlpChk_R | CTGTTCGACCATAGGAGTCATAGG |

F Genotyping results:

| Amplicon | Forward Primer | Position in WT | Position in KI, pre-Flp | Position in KI, post-Flp | Reverse Primer | Position in WT | Position in KI, pre-Flp | Position in KI, post-Flp | Size of Correct Amplicon (bp) | Result |
|---|---|---|---|---|---|---|---|---|---|---|
| **5' WT** | 5p_Comn_F | 397 | 397 | 397 | 5p_WT_R | 1326 | - | - | 930 | no amplicon |
| **5' KI** | 5p_Comn_F | 397 | 397 | 397 | 5p_KI_R | - | 1222 | - | 826 | 100% match by Sanger sequencing |
| **3' WT** | 3p_WT_F | 1101 | - | - | 3p_Comn_R | 1844 | - | - | 744 | no amplicon |
| **3' KI** | 3p_KI_F | - | 2749 | - | 3p_Comn_R | - | 3477 | - | 729 | 100% match by Sanger sequencing |
| **Flp Check** | FlpChk_F | - | - | 1100 | FlpChk_R | - | - | 1378 | 279 | 100% match by Sanger sequencing |

**Figure S1.** Overview of genotyping results confirming the successful introduction of one Csy4 DR into the 5' UTR of murine *CTSD* via CRISPR-Cas. **A**, Genomic structure of the mouse *CTSD* gene prior to modification, showing the locations of the gRNAs used for CRISPR-Cas (red). **B**, Design of the modified allele after integration of the targeting construct. Note the presence of a Puro$^r$ resistance cassette flanked by FRT sites (purple). **C**, Final structure of the modified CTSD allele after removal of the Puro$^r$ resistance cassette with Flp-recombinase. The approximate positions of genotyping primers are indicated in **A**-**C** (blue arrows). **D**, Table showing the gRNA sequences using for CRISPR-Cas, the targeted endogenous sequence (with PAM sequence in red), the amino-acid sequences encoded by the sequence targeted by gRNA_1, and the modifications to the corresponding targeted region within the KI targeting construct. Note that the top strand of the endogenous sequence is shown to highlight the translated region. **E**, Table showing the sequences for the DNA primers used for genotyping, with relative positions indicated in **A**-**C** (blue arrows). **F**, Summary of genotyping results, showing the relative positions of individual primers, the predicted amplicon sizes for primer pairs, and the outcome of PCR amplification and subsequent Sanger sequencing. These results apply to a single clonal cell line chosen for use in downstream experiments. Note that no amplification was obtained for the 5' or 3' WT amplicons.
